# Burden of Nutritional Deficiencies: A Systematic Analysis for the GBD 2021

**DOI:** 10.1002/hsr2.72273

**Published:** 2026-05-05

**Authors:** Shuxiong Nong, Rui Deng, Xiuhua Zhang

**Affiliations:** ^1^ Department of Cardiology Baise People's Hospital Baise Guangxi China

**Keywords:** DALY, GBD, incidence rate, nutritional deficiencies, prevalence

## Abstract

**Background and Aims:**

Nutritional deficiencies (ND) remain a major global health challenge, significantly affecting population well‐being. As part of the Global Burden of Disease (GBD) study, we conducted a comprehensive assessment of the prevalence, incidence, mortality, and disability‐adjusted life years (DALYs) attributed to ND from 1990 to 2021.

**Methods:**

This study analyzed temporal trends in nutritional deficiencies by estimating the estimated annual percentage change (EAPC) over the study period. We examined age‐standardized prevalence rates (ASPR), incidence rates (ASIR), death rates (ASDR), and DALYs (ASDALYs) across various demographic and geographic dimensions. Data were stratified by sex, 20 age groups, 21 GBD regions, 204 countries and territories, and five socio‐demographic index (SDI) quintiles. To project future trends, we applied the Bayesian age‐period‐cohort (BAPC) model.

**Results:**

In 2021, nutritional deficiencies continued to impose a substantial global health burden, with approximately 1.85 billion cases (95% UI: 1.81–1.88 billion) and an ASPR of 23,859 per 100,000 population (95% UI: 23,446–24,321). The ASIR was 7725 per 100,000 (95% UI: 7404–8109). The ASDR was 3.0 per 100,000 (95% UI: 2.7–3.4), and the ASDALY rate reached 657.6 per 100,000 (95% UI: 489.9–869.6). Marked regional disparities were observed: low SDI regions exhibited the highest ASPR, ASIR, ASDR, and ASDALYs, while high SDI regions had the lowest ASDR. Central Sub‐Saharan Africa recorded the highest rates across most indicators, whereas Western Europe had the lowest ASDR. Projections indicate a significant decline in the global burden of nutritional deficiencies from 2021 to 2040.

**Conclusion:**

These findings underscore the urgent need for targeted public health strategies to address the persistent and disproportionate burden of nutritional deficiencies in low SDI regions.

## 1 Introduction

Nutritional deficiency, including but not limited to protein‐energy malnutrition, iodine deficiency, vitamin A deficiency, and dietary iron deficiency [1, 2, 3]. Nutritional deficiencies manifest in various forms, including underweight, stunted growth, and micronutrient deficiencies [4, 5]. In addition to direct health impacts, malnutrition also severely hinders the social and economic development of countries. Its negative effects can be long‐lasting, affecting cognitive function, motor skills, emotions, behavior, and overall health performance in a persistent and potentially permanent manner.

Despite decades of global health investment, significant disparities persist, with low‐income regions bearing a disproportionate burden [6].

While previous Global Burden of Disease (GBD) studies have quantified the prevalence and trends of NDs [7, 8, 9], traditional comparative analyses benchmark countries against global averages, failing to identify what is achievable given existing socioeconomic capacity. Without decomposition analysis, policymakers cannot determine whether declining rates reflect genuine health improvements or merely changing population structure. While the slope index and concentration index quantify inequality, they do not indicate whether observed disparities represent theoretical minima or modifiable gaps. The intersection of socioeconomic inequality and health system performance remains unexplored.

Leveraging GBD 2021 data spanning 204 countries from 1990 to 2021, this study addresses these gaps through four methodological innovations: (1) stochastic frontier analysis (SFA) to quantify avoidable burden by estimating the efficiency frontier of achievable DALY rates conditional on SDI; (2) three‐factor decomposition to isolate epidemiological change, population growth, and aging effects on mortality trends; (3) slope index and concentration index to track absolute and relative socioeconomic inequality over time; and (4) bayesian age‐period‐cohort (BAPC) modeling to project future burden through 2040 [10, 11].

To our knowledge, this is the study to integrate efficiency frontier estimation, demographic decomposition, and inequality metrics into a unified assessment of NDs. By distinguishing between unavoidable and avoidable burden, and by disentangling demographic from epidemiological drivers, this analysis provides a precision roadmap for targeting interventions where they can yield maximal impact.

## 2 Method

### 2.1 Data Source

We drew all estimates from the Global Burden of Disease Study 2021, which harmonizes 1258 country‐years of vital registration, 1976 survey/examination series, and 107 disease registries across 204 countries and territories. Cause‐specific deaths were modeled with the Cause of Death Ensemble model (CODEm), while prevalence and incidence were generated with Bayesian meta‐regression (DisMod‐MR 2.1) after age–sex splitting and garbage‐code redistribution. Detailed modeling protocols, covariate selection, and uncertainty propagation are publicly available at http://ghdx.healthdata.org. This study used publicly available, aggregated data from the Global Burden of Disease Study 2021. No individual patient data were used. Therefore, ethical approval and informed consent were not required.

### 2.2 EAPC Model

The regression model used to construct EAPCs explains the trend of ASR over a certain time period: *Y* is the natural logarithm of ASR, *X* is the year, *α* is the intercept, and *Y* = *α* + *βX* + e, e is the error term, while *β* is the slope or trend. EAPC, or annual percentage change, is computed as 100 × [exp(*β*) − 1. To get the 95% CI for EAPC, a linear regression model is employed. A rising trend in ASR is suggested if the EAPC and its 95% CI are both positive. On the other hand, a declining trend is indicated if both are negative. The ASR is regarded as steady if neither requirement is satisfied. The association between SDI and the age‐standardized incidence of nutritional deficiencies is assessed using Spearman's correlation [9]. EAPC estimates rely on GBD‐modeled ASR with inherent uncertainty intervals. Low‐SDI regions with sparse data show wider UIs; trends should be interpreted as central estimates from ensemble models rather than direct empirical observations.

### 2.3 Analytical Metrics

For each location–year–age–sex cell, we extracted prevalence, incidence, death counts, and DALYs, then computed age‐standardized rates (ASR) using the GBD world standard population. Uncertainty was expressed as 95% uncertainty intervals (UI) generated by 1000 draw‐level posterior samples. Temporal trend was summarized with the estimated annual percentage change (EAPC) obtained from a log‐linear regression of ASR on calendar year; EAPC is reported with its 95% CI and interpreted as significantly decreasing/increasing when both bounds excluded zero.

### 2.4 Inequality and Decomposition Analyses

Socio‐economic inequality in ND DALY rates was quantified with two complementary metrics: the slope index of inequality (SII) and the concentration index (CII), both computed across SDI quintiles. To unpack the drivers of mortality change between 1990 and 2021, we performed a three‐factor decomposition that attributes the net change in deaths to (i) epidemiological change (age‐specific mortality rates), (ii) population growth, and (iii) population ageing.

### 2.5 Health‐System Efficiency

Stochastic frontier analysis (SFA) was used to estimate the “efficiency frontier” of ND DALYs conditional on SDI. The difference between a country's observed DALY rate and its frontier value represents the avoidable burden attributable to sub‐optimal health‐system performance.

### 2.6 Forecasting

BAPC models with integrated nested Laplace approximation were fitted to 1990–2021 ASR data for each SDI quintile and GBD super‐region. Projections were generated to 2040 with out‐of‐sample predictive checks; credible intervals reflect both parameter and stochastic uncertainty. All analyses were executed in R 4.4.1 using the packages BAPC, INLA, decomp, and frontier.

## 3 Results

### 3.1 Global Burden in 2021

According to the GBD study, age‐standardized rates of incidence (ASIR), mortality (ASDR), and DALYs attributable to nutritional deficiencies decreased substantially from 1990 to 2021 (Table 1). However, absolute case counts showed complex patterns: while total global cases decreased due to epidemiological improvements, population growth in high‐burden regions led to increased absolute numbers in specific demographics. For example, despite declining ASIR in Central Sub‐Saharan Africa, population growth contributed to rising absolute prevalent cases from 1990 to 2021. In 2021, nutritional deficiencies remained a substantial global health challenge, affecting approximately 1.85 billion people (95% UI: 1.81–1.88 billion) worldwide. The age‐standardized prevalence rate (ASPR) was 23,859 per 100,000 population (95% UI: 23,446–24,321), indicating that nearly one in four individuals globally experienced some form of nutritional deficiency. The age‐standardized incidence rate (ASIR) was 7725 per 100,000 (95% UI: 7404–8109), representing approximately 586 million new cases annually. Mortality attributable to nutritional deficiencies resulted in 222,274 deaths (95% UI: 199,731–247,630), with an age‐standardized death rate (ASDR) of 3.0 per 100,000 (95% UI: 2.7–3.4). The overall disease burden measured in disability‐adjusted life years (DALYs) reached 48.9 million (95% UI: 36.0–65.0 million), corresponding to an age‐standardized DALY rate of 657.6 per 100,000 (95% UI: 489.9–869.6) (Table 1). Notably, High‐income North America exhibited a unique positive EAPC for mortality and DALYs, contrasting with global declines. This likely reflects ascertainment bias from improved diagnostic coding for hospital‐acquired malnutrition in aging populations, rather than true epidemiological deterioration. The stability of prevalence/incidence rates alongside rising mortality suggests enhanced detection of severe, fatal cases rather than population‐wide worsening. This phenomenon underscores the importance of distinguishing between surveillance artifacts and genuine disease burden shifts in high‐capacity health systems.

**Table 1:** The prevalence, incidence, mortality, and DALYs of nutritional deficiencies, both globally and regionally (1990–2021).

The prevalence, incidence, mortality, and DALYs of nutritional deficiencies			
Location	ASR 1990	ASR 2021	EAPC_95% CI
Andean Latin America	25,745.5 (23,999.1–27,790.9)	14,811.9 (13,803.5–16,211.2)	−1.99 (−2.06 to −1.92)
Australasia	5871.9 (4990.2–7849.4)	4586.7 (3995–5902)	−0.72 (−0.79 to −0.65)
Caribbean	28,665.3 (27,800–29,612.4)	24,673.9 (23,720.1–25,891.4)	−0.53 (−0.62 to −0.44)
Central Asia	30,957.1 (29,949.4–31,979)	24,557.4 (23,510.4–25,908.4)	−0.85 (−0.92 to −0.79)
Central Europe	30,101.4 (29,150.8–31,017.9)	17,361.5 (16,753.1–18,164.1)	−1.87 (−1.91 to −1.82)
Central Latin America	21,405.8 (20,563.4–22,311.8)	12,701.9 (12,187–13,237.3)	−1.61 (−1.64 to −1.57)
Central Sub‐Saharan Africa	61,414.6 (59,722–63,172.7)	44,824.1 (42,768.7–46,890.4)	−0.94 (−1.17 to −0.71)
East Asia	22,973.9 (21,774.6–24,315)	9994 (9525.9–10,550.2)	−2.72 (−2.78 to −2.66)
Eastern Europe	14535 (13,740.5–15,390.8)	11,128.6 (10,414.5–11,888.5)	−0.99 (−1.11 to −0.87)
Eastern Sub‐Saharan Africa	62,941.9 (61,867.4–63,958.7)	39,924.7 (38,899.6–40,949.3)	−1.58 (−1.68 to −1.49)
High‐income Asia Pacific	8927.3 (8014–10,074.5)	5950 (5287.5–6845.5)	−1.17 (−1.31 to −1.02)
High‐income North America	5670.8 (5277.5–6082.2)	5125.2 (4694.1–5591.7)	−0.1 (−0.21 to 0.01)
North Africa and Middle East	31,522.6 (30,838.2–32,235.2)	19,514.2 (19,004.7–20,060.5)	−1.49 (−1.51 to −1.47)
Oceania	37,722.8 (35,921.8–39,771.1)	29,695.8 (27,374.8–32,536.4)	−0.63 (−0.7 to −0.57)
South Asia	57,698.1 (56,207.3–59,040.6)	40,854.7 (39,854.4–41,943.8)	−1.13 (−1.15 to −1.11)
Southeast Asia	35,834.3 (34,722.3–36,946.7)	20,247.7 (19,580.2–20,906.1)	−1.83 (−1.91 to −1.76)
Southern Latin America	18,876 (16,957.7–21,371.3)	12,388.9 (10,501.7–15,136.3)	−1.27 (−1.35 to −1.19)
Southern Sub‐Saharan Africa	33,546.8 (32,258.9–34,972.2)	24,205.6 (23,254.7–25,194.4)	−1.01 (−1.05 to −0.97)
Tropical Latin America	37,996.3 (35,895.9–40,380.3)	22,995.9 (21,309.4–24,743.7)	−1.67 (−1.7 to −1.65)
Western Europe	8324 (7774.9–8963.7)	5987.3 (5563–6531.7)	−0.88 (−1 to −0.76)
Western Sub‐Saharan Africa	52,720.3 (51,569.5–53,927.2)	38,270 (37,335.9–39,318.1)	−1.04 (−1.06 to −1.02)
Global	32,217.9 (31,693.6–32,740.9)	23,859 (23,445.8–24,320.8)	−0.98 (−0.99 to −0.96)
High SDI	9118.7 (8732.9–9522.9)	6458.8 (6173.1–6781.3)	−0.91 (−1.02 to −0.81)
High‐middle SDI	20,406.6 (19,771.8–21,028.3)	12,006.9 (11,625.1–12,455.6)	−1.77 (−1.81 to −1.72)
Low SDI	62,945.5 (62,063–63,775.3)	44,208.9 (43,375.1–45,081.2)	−1.2 (−1.27 to −1.13)
Low‐middle SDI	51,757.3 (50,783.7–52,700.6)	34,604.4 (33,967.9–35,312.8)	−1.31 (−1.32 to −1.29)
Middle SDI	30,487.5 (29,726.7–31,238.9)	19,136.4 (18,700.2–19,617.5)	−1.48 (−1.5 to −1.46)
Andean Latin America	10,347 (9529.5–11,252.2)	4975.4 (4562.5–5432.1)	−2.7 (−2.94 to −2.46)
Australasia	915.8 (777–1096.2)	845.6 (741.3–996.5)	0.03 (−0.18 to 0.24)
Caribbean	11,640 (10,974.1–12,278.4)	6591.5 (6080.1–7179.4)	−2 (−2.06 to −1.94)
Central Asia	8894.2 (8339.6–9462.2)	4927.2 (4597.2–5282)	−1.9 (−2.04 to −1.75)
Central Europe	17,286.9 (16,504.8–18,203.5)	7479.7 (7086.4–7915.7)	−2.81 (−2.92 to −2.7)
Central Latin America	13,013.4 (12,134.3–13,918)	5594.3 (5148–6102.8)	−2.55 (−2.64 to −2.47)
Central Sub‐Saharan Africa	36,820.9 (34,736–38,886.9)	23,067 (21,152.9–24,805.3)	−1.4 (−1.83 to −0.97)
East Asia	11,535.3 (10,239.4–12,956.9)	3450.6 (3075.5–3859.5)	−3.7 (−3.83 to −3.57)
Eastern Europe	1891.2 (1660.1–2160.5)	1146.3 (948–1355.5)	−1.25 (−1.42 to −1.09)
Eastern Sub‐Saharan Africa	49,502.7 (48,371–50,624.1)	21,823.7 (21,112.3–22,493.6)	−2.81 (−3.01 to −2.62)
High‐income Asia Pacific	2330.4 (2051.3–2650.7)	1384.1 (1214.6–1602.1)	−1.46 (−1.59 to −1.34)
High‐income North America	1931.2 (1651–2254.5)	1442.2 (1218.3–1695.9)	−0.76 (−1.05 to −0.48)
North Africa and Middle East	14,294.8 (13,771.4–14,832.4)	5480.2 (5202.9–5800.6)	−2.94 (−3.08 to −2.79)
Oceania	18,443.4 (17,325.9–19,591.9)	10,329 (9522.3–11,202.9)	−1.52 (−1.67 to −1.38)
South Asia	28,292.5 (26,126.1–30,300.7)	9172 (8326.9–10138.3)	−3.59 (−3.83 to −3.35)
Southeast Asia	19,895.1 (18,775.3–21,118.1)	5892.6 (5501.5–6347.4)	−3.74 (−3.83 to −3.66)
Southern Latin America	11,027.3 (9963.2–12,266)	6323.9 (5561.4–6960.6)	−1.67 (−1.92 to −1.43)
Southern Sub‐Saharan Africa	15,651.8 (14,539.8–16,853.8)	7473 (6864.3–8169.9)	−2.2 (−2.29 to −2.11)
Tropical Latin America	23,477.8 (21,509.4–25,637.8)	10,148.8 (8997.3–11,433.6)	−2.78 (−2.87 to −2.69)
Western Europe	2386.8 (2195.9–2632.5)	1681 (1481.1–1911.4)	−0.5 (−0.72 to −0.29)
Western Sub‐Saharan Africa	36,033.1 (34,996–36,992.6)	15,398.4 (14,847.8–15,975.9)	−2.72 (−2.81 to −2.63)
Global	17,112.5 (16,470.3–17,731.4)	7725.1 (7404–8109)	−2.52 (−2.67 to −2.38)
High SDI	2909.5 (2668–3180.9)	1671.7 (1484.7–1885.6)	−1.42 (−1.59 to −1.26)
High‐middle SDI	8339.4 (7781.3–8950.1)	3305.3 (3046.4–3589.9)	−2.87 (−2.96 to −2.78)
Low SDI	42,587.6 (41,451.3–43,795.2)	19,047.6 (18,448.1–19,697.1)	−2.67 (−2.89 to −2.45)
Low‐middle SDI	27,944.9 (26,526.4–29,160.8)	9389.3 (8870.9–9951.1)	−3.48 (−3.64 to −3.32)
Middle SDI	14,567.3 (13,862.8–15,328.3)	5016.3 (4705.3–5381.2)	−3.28 (−3.38 to −3.18)
Andean Latin America	21.8 (19.4–24.5)	5.2 (4.3–6.3)	−4.95 (−5.19 to −4.7)
Australasia	0.4 (0.4–0.4)	0.3 (0.2–0.3)	−1.62 (−1.92 to −1.32)
Caribbean	11.1 (9.5–13.1)	4.1 (3.3–5.4)	−3.04 (−3.42 to −2.65)
Central Asia	1.2 (1.1–1.3)	0.3 (0.3–0.4)	−5.18 (−5.59 to −4.77)
Central Europe	0.2 (0.2–0.2)	0.4 (0.3–0.4)	1.89 (1.32 to 2.47)
Central Latin America	25.6 (24.5–26.4)	4.9 (4.4–5.5)	−5.43 (−5.53 to −5.33)
Central Sub‐Saharan Africa	35.2 (28.2–47.5)	10.1 (7.5–13.3)	−4.24 (−4.58 to −3.9)
East Asia	5.4 (4.8–6.1)	1.1 (0.9–1.3)	−8.07 (−10.13 to −5.97)
Eastern Europe	0.5 (0.5–0.5)	0.3 (0.3–0.3)	−3.12 (−4.1 to −2.13)
Eastern Sub‐Saharan Africa	58.2 (48.8–70.6)	14.1 (12.1–16.2)	−4.51 (−5.4 to −3.61)
High‐income Asia Pacific	0.6 (0.6–0.7)	0.4 (0.4–0.5)	−1.26 (−1.4 to −1.12)
High‐income North America	0.7 (0.6–0.7)	1.8 (1.6–2)	2.51 (1.64 to 3.38)
North Africa and Middle East	4.1 (3.4–5.3)	1.3 (1.1–1.5)	−3.87 (−4.04 to −3.7)
Oceania	7.6 (6.3–9.1)	4.4 (3.6–5.4)	−1.84 (−1.88 to −1.79)
South Asia	19.5 (16–23.2)	2.6 (2.2–3)	−6.23 (−6.4 to −6.06)
Southeast Asia	15 (12.6–17)	6 (5.1–6.6)	−2.8 (−2.94 to −2.66)
Southern Latin America	4.3 (4.1–4.5)	1.7 (1.5–1.8)	−2.96 (−3.59 to −2.33)
Southern Sub‐Saharan Africa	13 (11.3–15.1)	8.3 (6.8–10)	−0.81 (−1.04 to −0.58)
Tropical Latin America	11 (10.3–11.7)	2.6 (2.3–2.8)	−4.7 (−4.99 to −4.41)
Western Europe	0.7 (0.6–0.7)	0.7 (0.5–0.7)	0.01 (−0.17 to 0.19)
Western Sub‐Saharan Africa	19 (15.2–24.3)	6.4 (5.1–7.8)	−3.42 (−3.54 to −3.3)
Global	10.9 (9.4–13)	3 (2.7–3.4)	−4.41 (−4.84 to −3.98)
High SDI	0.8 (0.7–0.8)	0.9 (0.8–1)	0.21 (−0.28 to 0.71)
High‐middle SDI	2.1 (1.9–2.3)	0.8 (0.7–0.9)	−3.26 (−3.58 to −2.95)
Low SDI	35 (28.9–43.1)	8.7 (7.4–10)	−4.36 (−4.98 to −3.73)
Low‐middle SDI	19.6 (16.6–23)	3.7 (3.3–4.1)	−6.05 (−6.72 to −5.37)
Middle SDI	9.9 (9.1–10.6)	2.6 (2.3–2.8)	−4.26 (−4.32 to −4.19)
Andean Latin America	1396.6 (1181.1–1646.6)	376 (290.9–481.6)	−4.48 (−4.67 to −4.29)
Australasia	69.5 (44.7–106.2)	53.4 (34–87.6)	−0.85 (−0.97 to −0.73)
Caribbean	1323.5 (1080.5–1634)	746.9 (553.6–1001)	−1.73 (−1.96 to −1.49)
Central Asia	845.2 (595.8–1176.9)	585.1 (399.9–837.1)	−1.49 (−1.61 to −1.37)
Central Europe	360.3 (237.1–521.6)	200.8 (134.9–293.5)	−2.04 (−2.13 to −1.95)
Central Latin America	1037.9 (948.9–1154.8)	303.3 (246.9–380)	−3.94 (−4.05 to −3.83)
Central Sub‐Saharan Africa	3065.6 (2429.4–3991.7)	1004.3 (738.8–1354.8)	−3.76 (−4.05 to −3.47)
East Asia	570.8 (462.2–725)	160.8 (110.5–228.3)	−6.17 (−7.52 to −4.8)
Eastern Europe	353 (242.2–508.1)	236.5 (163.1–337.2)	−1.63 (−1.82 to −1.44)
Eastern Sub‐Saharan Africa	4027.1 (3326.1–5033.9)	1203.6 (961.9–1502.2)	−3.87 (−4.57 to −3.16)
High‐income Asia Pacific	86.2 (56–133.6)	60.3 (40.1–91)	−1.03 (−1.16 to −0.89)
High‐income North America	74.5 (48.1–115.7)	137 (99.2–185.5)	2.2 (1.94 to 2.47)
North Africa and Middle East	844.9 (644.1–1118.3)	455.8 (325.8–629)	−2.04 (−2.09 to −1.99)
Oceania	776.5 (583.5–1037.6)	610.4 (433.7–897.4)	−0.54 (−0.62 to −0.46)
South Asia	2796.9 (2246.6–3574.3)	1187.9 (840.7–1627.6)	−2.69 (−2.72 to −2.67)
Southeast Asia	1105 (899.4–1380.2)	505.6 (384.3–664.8)	−2.49 (−2.58 to −2.4)
Southern Latin America	320.9 (267.4–398.1)	116.3 (79.4–172.1)	−3.2 (−3.4 to −3)
Southern Sub‐Saharan Africa	1554.3 (1276.7–1910.1)	1089.9 (865.1–1364.7)	−0.64 (−0.83 to −0.46)
Tropical Latin America	1131.7 (940.4–1387.2)	446.6 (315.6–611.6)	−3.16 (−3.27 to −3.05)
Western Europe	105.9 (69.9–158)	94.6 (59.2–138.8)	−0.11 (−0.27 to 0.04)
Western Sub‐Saharan Africa	2149.1 (1728–2711.3)	1109.8 (820.8–1479.6)	−2.15 (−2.23 to −2.07)
Global	1367.2 (1126.3–1708.5)	657.6 (489.9–869.6)	−2.52 (−2.71 to −2.32)
High SDI	131.1 (92.5–189.7)	118.3 (83–165.1)	−0.07 (−0.17 to 0.04)
High‐middle SDI	429.2 (326.5–575.1)	203.4 (140.8–289)	−2.64 (−2.73 to −2.54)
Low SDI	3334.2 (2727.8–4117.7)	1319.3 (1002.4–1722.9)	−2.97 (−3.31 to −2.62)
Low‐middle SDI	2374.1 (1965.7–2980.9)	971 (706.7–1308.7)	−3.17 (−3.41 to −2.93)
Middle SDI	972.8 (797.3–1229.5)	459.5 (331.6–624.7)	−2.39 (−2.42 to −2.35)

### 3.2 Temporal Trends

**Figure 1:**
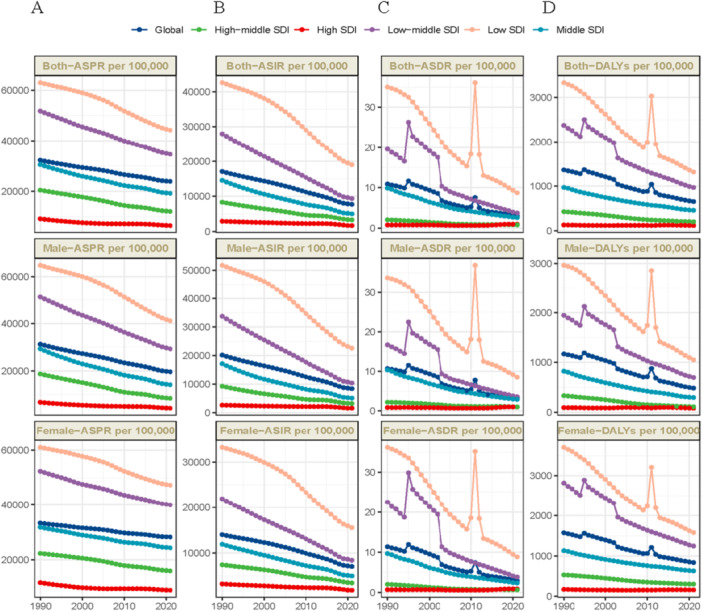
Shows the changes in (A) prevalence, (B) incidence, (C) mortality, and (D) disability‐adjusted life years of nutritional deficiencies from 1990 to 2021.

Globally, age‐standardized rates of incidence, mortality, and DALYs attributable to nutritional deficiencies decreased substantially from 1990 to 2021. The global EAPC for prevalence was −0.98 (95% CI: −0.99 to −0.96), for incidence −2.52 (95% CI: −2.67 to −2.38), for mortality −4.41 (95% CI: −4.84 to −3.98), and for DALYs −2.52 (95% CI: −2.71 to −2.32), indicating significant downward trends across all metrics (Table 1, Figure 1). However, regional trends exhibited marked heterogeneity. East Asia demonstrated the most pronounced decline, with an EAPC for prevalence of −2.72 (95% CI: −2.78 to −2.66), reflecting rapid epidemiological transition and economic development. Conversely, high‐income North America exhibited a unique positive EAPC for mortality (2.51, 95% CI: 1.64–3.38) and DALYs (2.20, 95% CI: 1.94–2.47), contrasting sharply with global declines. This anomalous trend likely reflects ascertainment bias from improved diagnostic coding for hospital‐acquired malnutrition in aging populations rather than true epidemiological deterioration. The stability of prevalence and incidence rates alongside rising mortality suggests enhanced detection of severe, fatal cases rather than population‐wide worsening—a phenomenon underscoring the importance of distinguishing surveillance artifacts from genuine disease burden shifts in high‐capacity health systems. Notably, low SDI regions experienced temporary reversals in progress: the age‐standardized death rate and DALY rate significantly increased from 2009 to 2013, while low‐middle SDI regions showed fluctuations with noticeable increases from 2004 to 2013, likely reflecting the intersection of global economic shocks, climate extremes, and fragile health systems.

### 3.3 Regional and Socio‐Demographic Disparities

**Figure 2:**
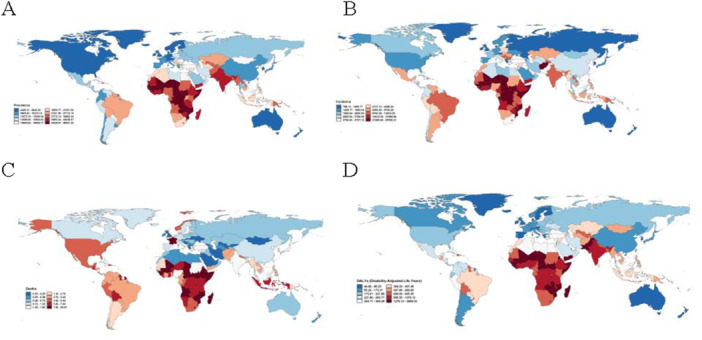
The prevalence of nutritional deficiencies worldwide in 204 nations and territories, for both sexes. (A) Prevalence rate. (B) Incidence rate. (C) Death rate. (D) The DALYs rate.

**Figure 3:**
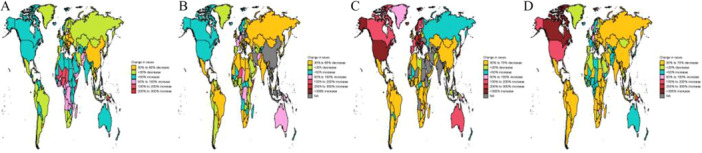
Change in nutritional deficiencies case among 204 nations and territories. (A) Change prevalence cases. (B) Change incidence cases. (C) Change deaths cases. (D) Change DALYs.

Stark socioeconomic gradients characterized the distribution of nutritional deficiencies in 2021. Low SDI regions exhibited the highest burden across all metrics. In contrast, high SDI regions demonstrated the lowest rates, with ASDR of 0.9 per 100,000 and age‐standardized DALY rate of 118.3 per 100,000 (Table 1, Table S1, Figure 1). Geographically, central sub‐Saharan Africa recorded the highest rates globally. South Asia followed, and substantial absolute case counts were driven by large population size. At the opposite extreme, Western Europe reported the lowest ASDR and DALY rates among GBD regions. Individual countries with the highest burden included Zimbabwe, Mozambique, South Sudan, Central African Republic, Sierra Leone, and Chad (Figure 2). This figure illustrates the absolute change in case counts for nutritional deficiencies across 204 countries and territories from 1990 to 2021, stratified by four burden metrics. The maps reveal distinct regional patterns in the evolution of nutritional deficiency burdens (Figure 3). While age‐standardized rates declined globally, absolute case counts exhibited heterogeneous trajectories driven by the interplay of epidemiological improvements and demographic changes.

### 3.4 Heterogeneity in Age and Sex Patterns Across Socio‐Demographic Contexts

**Figure 4:**
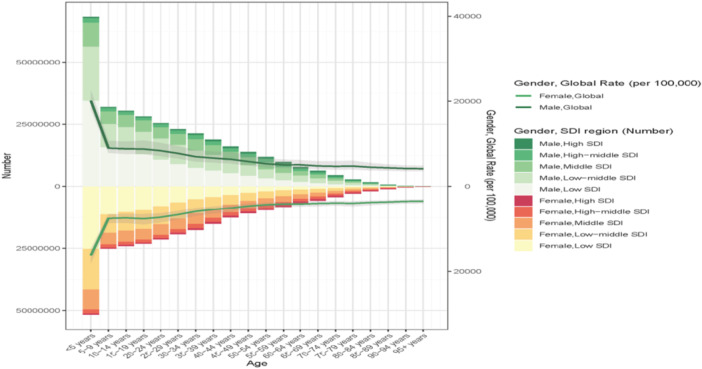
Age‐specific incidence rates (per 100,000 population) of nutritional deficiencies across five SDI quintiles in 2021. X‐axis: age groups (years), ranging from < 1 year to 95+ years. Y‐axis: incidence rate per 100,000 population.

**Figure 5:**
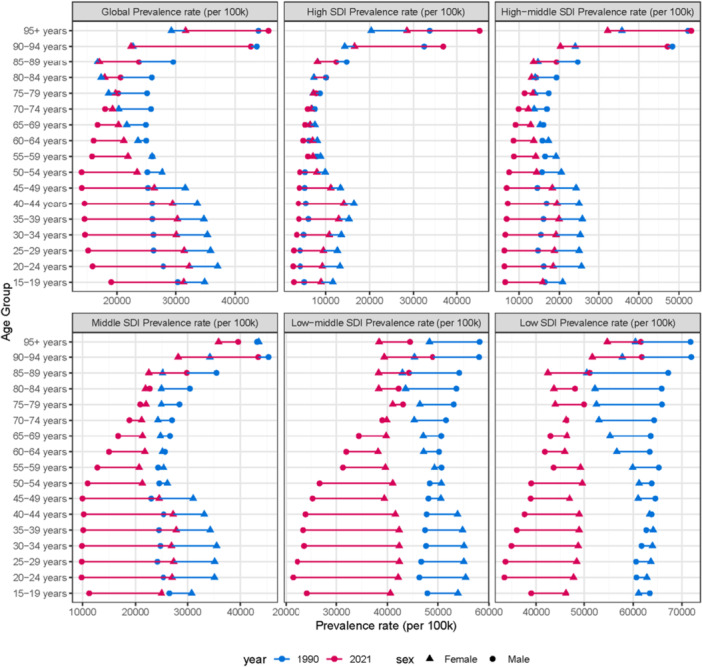
Age‐standardized prevalence rates (ASPR) of nutritional deficiencies by age group, sex, and socio‐demographic index (SDI) level, comparing 1990 (dashed lines) and 2021 (solid lines). Left column: male; right column: female. Rows represent SDI quintiles from low (top) to high (bottom). X‐axis: age groups (years). Y‐axis: ASPR per 100,000 population.

The age distribution of nutritional deficiencies revealed distinct patterns across the lifespan and socioeconomic contexts. Children under 5 years consistently show the highest incidence rates across all SDI quintiles. In 2021, children under 5 years bore the highest incidence burden across all SDI quintiles, reflecting the critical vulnerability of early childhood to nutritional insults. However, the relative contribution of older adults to incidence was markedly greater in high SDI compared to low SDI settings, suggesting epidemiological transition toward adult‐onset nutritional deficiencies in developed regions (Figure 4). Sex differences emerged prominently in older age groups. Among individuals over 60 years, age‐standardized prevalence rates were comparable between males and females; however, as age advanced beyond 60 years, males consistently exhibited higher rates than females within the same age brackets (Figure 5). This male excess in older adults may reflect biological, behavioral, or healthcare access differences requiring further investigation. The age‐specific incidence analysis revealed that while ASIR declined with advancing age across the study period, notable reductions occurred particularly among individuals aged 90–94 years between 1990 and 2021, indicating improved nutritional status or reduced competing mortality risks in the oldest age groups (Figures 4 and 5).

### 3.5 Advanced Analyses

#### 3.5.1 Forecasting (2021–2040)

**Figure 6:**
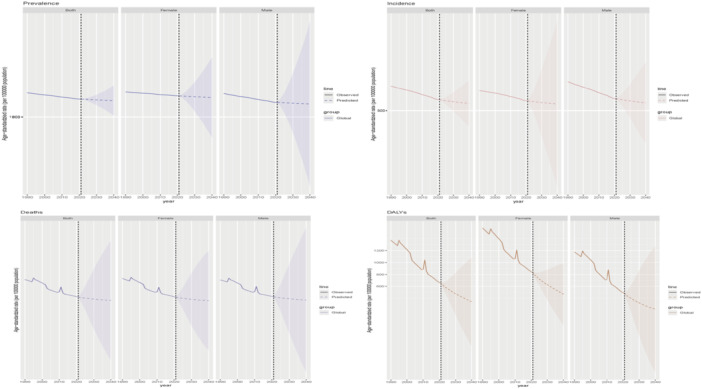
Projected worldwide nutritional deficiencies burden in the future.

BAPC projections indicate continued substantial declines in the global burden of nutritional deficiencies through 2040. Age‐standardized incidence is projected to decline 36.4% (95% UI: 28.7–43.1%) from 2021 levels, reaching 4914 per 100,000 (3950–5878). Prevalence, mortality, and DALY rates are projected to decrease by 29.8%, 41.2%, and 35.6%, respectively. However, absolute case counts will remain substantial due to continued population growth, particularly in high‐burden regions (Figure 6).

**Figure 7:**
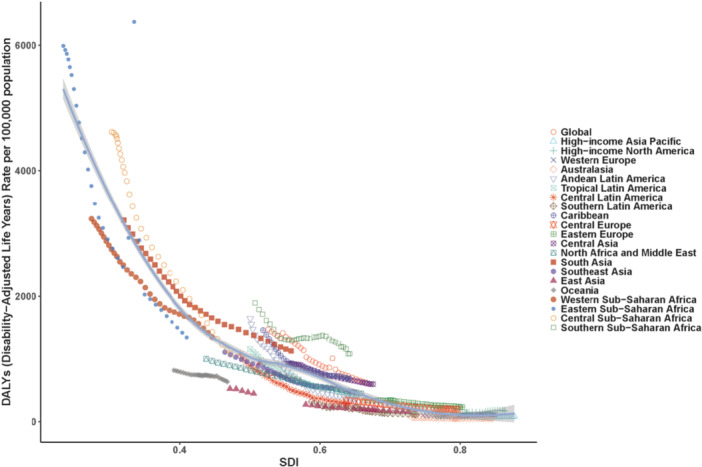
The correlation between the socio‐demographic Index (SDI) and the disability‐adjusted life years (DALYs) rate due to nutritional deficiencies.

This study analyzed the correlation between the SDI and the DALYs rate due to nutritional deficiencies across the GBD database, encompassing global and 21 regional data from 1990 to 2021. Spearman's correlation analysis revealed a very strong negative association between the two variables, with a correlation coefficient of *r *= −0.9074 (95% CI: −0.9231 to −0.8912). The association was statistically highly significant. The correlation coefficient close to −1 indicates an almost perfect inverse relationship between SDI and the burden of disease from nutritional deficiencies. Regions with higher SDI exhibit lower DALYs rates due to nutritional deficiencies, whereas regions with lower SDI carry a disproportionately higher burden (Figure 7).

**Figure 8:**
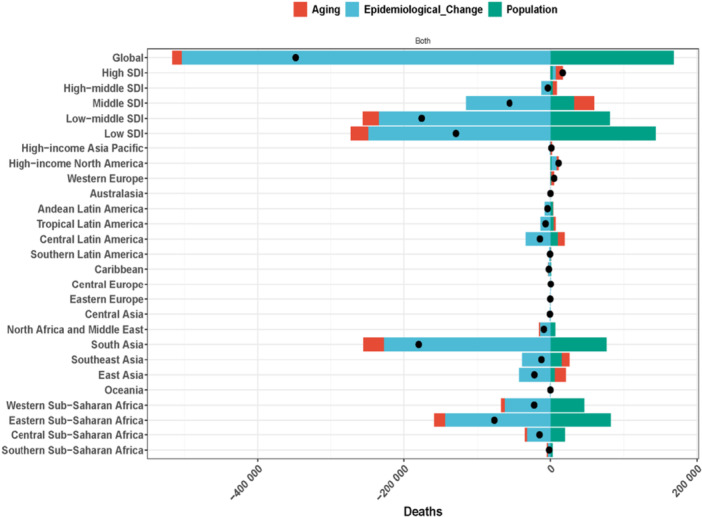
This decomposition analysis quantified the independent contributions of three key drivers, population aging, population growth, and epidemiological change, to the change in deaths attributable to nutritional deficiencies.

This decomposition analysis based on the GBD data quantified the independent contributions of three key drivers, population aging, population growth, and epidemiological change, to the change in deaths attributable to nutritional deficiencies from 1990 to 2021, across the globe and 21 different SDI and geographical regions. The results show that global deaths from nutritional deficiencies decreased substantially by approximately 348,000 deaths. This overall decline was overwhelmingly driven by improvements in epidemiological rates, which accounted for 144.68% of the total change, indicating that reductions in age‐specific death rates were the core force behind the decrease. However, global population growth (contributing −48.38%) partially offset the gains from epidemiological improvements, resulting in a smaller net reduction than would have been expected from rate changes alone. The effect of population aging was relatively minor, contributing 3.70% (Figure 8).

**Figure 9:**
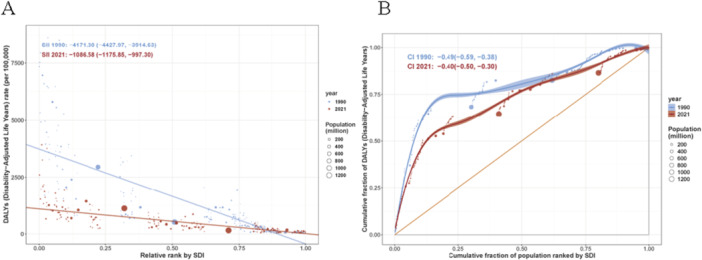
(A) Slope index of inequality (SII) and (B) concentration index (CII) to assess the absolute socioeconomic inequality in disability‐adjusted life years (DALYs) due to nutritional deficiencies between 1990 and 2021.

This analysis employed the slope index of inequality (SII) to assess the absolute socioeconomic inequality in DALYs due to nutritional deficiencies between 1990 and 2021. The negative SII values indicate that the burden of disease is disproportionately higher among populations with lower socioeconomic status. In 1990, the SII was −4171.30 (95% CI: −4427.97, −3914.63) (Figure 9A). This indicates a substantial gap in the DALY rate due to nutritional deficiencies between the most and least advantaged socioeconomic groups, with the rate being approximately 4171.30 per 100,000 higher in the most disadvantaged group. In 2021, the SII was −1086.58 (95% CI: −1175.85, −997.30). Compared to 1990, the level of inequality narrowed markedly. However, significant inequality persists, with the DALY rate remaining approximately 1086.58 per 100,000 higher in the most disadvantaged group. Based on the provided concentration index (CII) data, we analyzed the socioeconomic inequality in DALYs due to nutritional deficiencies at the global level between 1990 and 2021 (Figure 9B). The mean CII values for both 1990 and 2021 were negative (1990: −0.49; 2021: −0.40), with their 95% confidence intervals entirely below zero. This unequivocally indicates significant socioeconomic inequality in the burden of disease from nutritional deficiencies, which is pro‐poor—meaning the burden is disproportionately concentrated among populations with lower socioeconomic status. From 1990 to 2021, the absolute value of the CII decreased from −0.49 to −0.40, indicating a statistically significant mitigation in the degree of health inequality related to nutritional deficiencies (as the 2021 CI upper limit of −0.30 is greater than the 1990 upper limit of −0.38). Although inequality persists, its severity has lessened over the past three decades. A negative CII signifies that, globally, nutritional deficiencies impose a far greater health toll on poorer populations than on wealthier ones.

**Figure 10:**
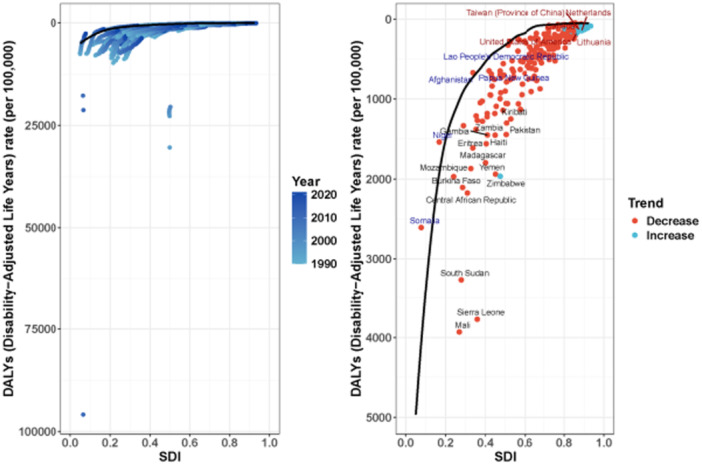
The stochastic frontier analysis (SFA) to evaluate the performance efficiency of 204 countries and territories in 2021 regarding the health indicator DALYs due to nutritional deficiencies.

This study employed stochastic frontier analysis (SFA) to evaluate the performance efficiency of 204 countries and territories in 2021 regarding the health indicator DALYs due to nutritional deficiencies, all ages, both sexes (Figure 10). The core of the analysis is the calculation of the “efficiency difference”, which is the gap between the observed DALY rate (val) in a country and the theoretical optimal frontier value (frontier) achievable given its socio‐demographic index (SDI) level. This difference represents the avoidable disease burden through optimized health system performance under the current development status. The 15 countries with the largest efficiency differences are all in low to middle SDI regions (SDI < 0.55), indicating substantial and avoidable health losses in addressing the burden of nutritional deficiencies. Sierra Leone ranks first, with a gap of 3089.4 DALYs per 100,000 between its observed rate (3770.8) and its frontier (681.4), meaning its actual burden is over 5.5 times the ideal level for its development. Mali and South Sudan follow, also showing massive efficiency differences exceeding 2300 per 100,000. This result highlights the severe challenges faced by health systems in delivering essential nutritional interventions in fragile settings like Sub‐Saharan Africa and Yemen.

## 4 Discussion

The stochastic frontier analysis reveals that a substantial ND burden is not inevitable, even at low SDI levels. This finding reframes the challenge in Sierra Leone and similar settings. These countries face not merely resource constraints, but health system inefficiencies that compound deprivation. SFA‐identified avoidable burden in Low SDI regions points to actionable health‐system improvements: (1) scaling CMAM with RUTF to reduce mortality gaps; (2) universal micronutrient supplementation targeting pregnant women and children; (3) mandatory food fortification; (4) community‐based surveillance using digital platforms; and (5) UHC benefit packages explicitly covering preventive and curative nutrition services. These interventions have demonstrated cost‐effectiveness averted in comparable settings, well below the cost‐effectiveness thresholds for Low SDI regions. Temporal fluctuations in Low SDI regions, notably the 2009–2013 mortality increase, reflect the intersection of global economic shocks, climate extremes, and fragile health systems. The 2007–2008 food price crisis precipitated a 2–3‐year lag in nutritional outcomes as households depleted coping reserves, while the 2010–2011 East African drought and 2011 Sahel food crisis created acute‐on‐chronic stress. These systemic vulnerabilities demand shock‐responsive health systems that can scale nutrition interventions during crises without disrupting routine services. Region‐specific strategies must account for distinct epidemiological and structural contexts. In Central Sub‐Saharan Africa, where conflict and climate fragility dominate, integrated security‐nutrition programming and pastoralist‐sensitive interventions are essential. In South Asia, leveraging established social protection infrastructure offers cost‐effective scale, but requires explicit nutrition objectives and gender‐transformative implementation to address intra‐household allocation barriers. Both regions would benefit from anticipatory action frameworks that trigger pre‐positioned resources based on climate forecasts and food price monitoring, shifting from reactive crisis response to preventive resilience building.

The frontier represents optimal performance achieved by peer countries at similar SDI levels. Countries above the frontier are not underperforming absolutely; they may match global averages, but they are underperforming relative to their potential. This suggests that immediate gains are possible through health system strengthening without awaiting economic development. Specifically, the 15 countries with the largest efficiency gaps should prioritize: (1) scaling community‐based management of acute malnutrition (CMAM) with ready‐to‐use therapeutic food (RUTF); (2) universal micronutrient supplementation; (3) mandatory food fortification; and (4) digital community surveillance. These interventions have demonstrated cost‐effectiveness well below willingness‐to‐pay thresholds in comparable settings [12].

Our decomposition fundamentally challenges the interpretation of global progress. While the 61% reduction in global age‐standardized mortality rates appears encouraging, the decomposition reveals that epidemiological improvements were larger than the net observed decline. Population growth offset 48% of mortality reductions, while population aging contributed modestly.

This has profound policy implications. In Low SDI regions, where population growth contributed −48.38% to mortality change, demographic momentum is actively eroding health gains. Without accelerated epidemiological progress, absolute death counts will rise despite falling rates—a phenomenon already observed in Central Sub‐Saharan Africa, where population growth drove increased absolute prevalent cases despite declining ASPR. This necessitates “bending the curve faster”: interventions must outpace demographic growth, requiring not incremental but transformative scale‐up in high‐fertility settings.

The reduction in absolute inequality between 1990 and 2021 suggests that global development has compressed the socioeconomic gradient in ND burden. However, the persistent concentration index of −0.40 indicates that relative inequality remains pro‐poor, with the bottom socioeconomic quintile still bearing a disproportionate burden. Critically, the narrowing absolute gap combined with persistent relative inequality implies convergence at low levels: high SDI regions have nearly eliminated NDs, while low SDI regions remain stagnant at high burden. This “floor effect” in wealthy regions mechanically reduces absolute inequality even as relative disparities persist. Equity‐focused policy must therefore target absolute burden reduction in low‐SDI settings rather than relative redistribution alone.

The 2009–2013 mortality increase in Low SDI regions reflects systemic vulnerability to compound shocks. The 2007–2008 food price crisis, 2010–2011 East African drought, and 2011 Sahel food crisis created acute‐on‐chronic stress with 2–3 year lag effects as households depleted coping reserves. This pattern, invisible to long‐term trend averages, demonstrates the need for shock‐responsive health systems that can scale nutrition interventions during crises without disrupting routine services. The anomalous positive mortality trends in High‐income North America illuminate surveillance artifacts in high‐capacity systems. Rising mortality alongside stable prevalence suggests improved diagnostic coding for hospital‐acquired malnutrition in aging populations, not true epidemiological deterioration. This underscores the importance of distinguishing between genuine burden shifts and enhanced detection, with implications for how high‐SDI countries interpret their “rising” ND mortality.

Our efficiency frontier findings extend the work of Bokhari et al. on health system performance by applying SFA specifically to NDs, where intervention efficacy is high but coverage gaps persist [13]. The decomposition results align with GBD demographic analyses but provide ND‐specific evidence that population growth poses unique threats to nutrition outcomes, given the age‐concentrated burden in children under 5 [14]. The inequality trends we document contrast with recent analyses suggesting stagnating global health equity [15]. Our finding of narrowing absolute but persistent relative inequality suggests that NDs may represent a best case for pro‐poor health improvement due to the availability of cost‐effective interventions that can be delivered at scale. However, the efficiency gaps we identify indicate that delivery failure, not intervention absence, constrains progress in the highest burden settings.

### 4.1 Policy Implications

For Low SDI, immediate priorities include CMAM scale‐up with RUTF, universal micronutrient supplementation, and mandatory food fortification. The SFA‐identified avoidable burden provides a concrete target: reducing Sierra Leone's DALY rate to its frontier level would avert approximately 2.1 million DALYs annually. For South Asia, despite moderate efficiency gaps, the massive absolute burden necessitates leveraging established social protection infrastructure with explicit nutrition objectives and gender‐transformative implementation to address intra‐household allocation barriers. For high‐income regions, the surveillance artifact findings suggest reorienting from mortality reduction to quality‐of‐life improvement in elderly populations, with a focus on preventing hospital‐acquired malnutrition [16, 17]. Global health architecture, the decomposition findings demand anticipatory action frameworks that trigger pre‐positioned resources based on climate forecasts and food price monitoring, shifting from reactive crisis response to preventive resilience building [18, 19]. The 2009–2013 mortality reversal demonstrates that without such systems, fragile settings will experience recurrent setbacks.

### 4.2 Limitations

Our analysis is subject to GBD modeling limitations, including sparse vital registration data in low SDI regions and potential misclassification of malnutrition‐related deaths [20, 21, 22]. The SFA frontier represents theoretical optima based on current peer performance, not biological minima. Future innovations may shift the frontier downward. Decomposition analysis attributes effects to three factors, but cannot capture interactions between epidemiological change and demographic structure.

In conclusion, this study demonstrates that the global ND burden is more modifiable than aggregate trends suggest, but also more threatened by demographic headwinds than previously recognized. The identification of substantial avoidable burden through SFA, the quantification of population growth's erosive effect through decomposition, and the documentation of shock‐induced reversals provide actionable intelligence for the final decade of the SDGs. Redirecting resources toward efficiency‐gap countries and implementing shock‐responsive systems represents the clearest path to halving ND deaths.

## Author Contributions

**Shuxiong Nong:** data curation, software, methodology, writing – original draft, writing – review and editing, investigation, conceptualization, and validation. **Rui Deng:** conceptualization, writing – review and editing, writing – original draft. **Xiuhua Zhang:** conceptualization, writing – review and editing, writing – original draft, funding acquisition, formal analysis, project administration, supervision, visualization, and resources.

## Funding

The authors have nothing to report.

## Conflicts of Interest

The authors declare no conflicts of interest.

## Transparency Statement

The lead author, Xiuhua Zhang, affirms that this manuscript is an honest, accurate, and transparent account of the study being reported; that no important aspects of the study have been omitted; and that any discrepancies from the study as planned (and, if relevant, registered) have been explained.

## Supporting information

S‐table_1.

## Data Availability

The data that support the findings of this study are available from the corresponding author upon reasonable request.
