# Supplementary material for: Burden of Nutritional Deficiencies: A Systematic Analysis for the GBD 2021
Source: Health Sci Rep. 2026 May 5;9(5):e72273. doi: 10.1002/hsr2.72273 (PMC13140514; doi:10.1002/hsr2.72273)
Supplement: Supplementary file 1 — S‐table_1. [file HSR2-9-e72273-s001.docx]

**The prevalence, incidence, mortality, and DALYs of nutritional deficiencies**

| **Prevalence** | | | | | |
| --- | --- | --- | --- | --- | --- |
| **location** | **Number 1990** | **ASR 1990** | **Number 2021** | **ASR 2021** | **EAPC_95%CI** |
| Andean Latin America | 10484115.1 (9725704.3-11391468.1) | 25745.5 (23999.1-27790.9) | 9760589.8 (9070653.5-10669802.6) | 14811.9 (13803.5-16211.2) | -1.99 (-2.06 to -1.92) |
| Australasia | 1158776.4 (1010769.6-1459269.8) | 5871.9 (4990.2-7849.4) | 1459598 (1318169.4-1709488.7) | 4586.7 (3995-5902) | -0.72 (-0.79 to -0.65) |
| Caribbean | 10377560.7 (10049815.2-10735795.7) | 28665.3 (27800-29612.4) | 11389503.4 (10962545.6-11940346.3) | 24673.9 (23720.1-25891.4) | -0.53 (-0.62 to -0.44) |
| Central Asia | 22334239.3 (21598359-23179108) | 30957.1 (29949.4-31979) | 23804196.7 (22785372.5-25092730.6) | 24557.4 (23510.4-25908.4) | -0.85 (-0.92 to -0.79) |
| Central Europe | 36725050.7 (35598638.1-37811773.8) | 30101.4 (29150.8-31017.9) | 19224721.5 (18602276.4-20012742.5) | 17361.5 (16753.1-18164.1) | -1.87 (-1.91 to -1.82) |
| Central Latin America | 37544532.7 (35853779-39239515.5) | 21405.8 (20563.4-22311.8) | 31383870.2 (30058891.2-32681022) | 12701.9 (12187-13237.3) | -1.61 (-1.64 to -1.57) |
| Central Sub-Saharan Africa | 34424749.5 (33307032.8-35637672.9) | 61414.6 (59722-63172.7) | 61913266.3 (58993967.2-65101628.2) | 44824.1 (42768.7-46890.4) | -0.94 (-1.17 to -0.71) |
| East Asia | 281133227.2 (265234703.5-298969903.2) | 22973.9 (21774.6-24315) | 152264619 (144553425.3-160840133) | 9994 (9525.9-10550.2) | -2.72 (-2.78 to -2.66) |
| Eastern Europe | 32667545.7 (30870116.5-34777511.7) | 14535 (13740.5-15390.8) | 23276070.9 (21637366.3-25071286) | 11128.6 (10414.5-11888.5) | -0.99 (-1.11 to -0.87) |
| Eastern Sub-Saharan Africa | 121213649.3 (118849394-123447869.7) | 62941.9 (61867.4-63958.7) | 170733559.6 (166269936.3-175406206.9) | 39924.7 (38899.6-40949.3) | -1.58 (-1.68 to -1.49) |
| High-income Asia Pacific | 15173415.3 (13504296-17218684.3) | 8927.3 (8014-10074.5) | 12515713.8 (11273662.2-14208105.8) | 5950 (5287.5-6845.5) | -1.17 (-1.31 to -1.02) |
| High-income North America | 16298514.5 (15102005.5-17479237.7) | 5670.8 (5277.5-6082.2) | 20313301.7 (18658728.7-22271771.7) | 5125.2 (4694.1-5591.7) | -0.1 (-0.21 to 0.01) |
| North Africa and Middle East | 110410392.3 (107908788.8-113281399.3) | 31522.6 (30838.2-32235.2) | 120972869.1 (117686882.9-124467697.5) | 19514.2 (19004.7-20060.5) | -1.49 (-1.51 to -1.47) |
| Oceania | 2551813.8 (2411741.6-2721367) | 37722.8 (35921.8-39771.1) | 4248613.2 (3881670.3-4672494.7) | 29695.8 (27374.8-32536.4) | -0.63 (-0.7 to -0.57) |
| South Asia | 634104800.1 (617323641-650116132.7) | 57698.1 (56207.3-59040.6) | 739051761 (719938199.7-759461377.2) | 40854.7 (39854.4-41943.8) | -1.13 (-1.15 to -1.11) |
| Southeast Asia | 172103477.2 (166521018.5-178061547.8) | 35834.3 (34722.3-36946.7) | 137595401.5 (133035023.1-142160406.3) | 20247.7 (19580.2-20906.1) | -1.83 (-1.91 to -1.76) |
| Southern Latin America | 9387603.9 (8412554.8-10661326) | 18876 (16957.7-21371.3) | 7990908.2 (6837460.3-9742035.1) | 12388.9 (10501.7-15136.3) | -1.27 (-1.35 to -1.19) |
| Southern Sub-Saharan Africa | 19125679.5 (18322311.6-20066054.7) | 33546.8 (32258.9-34972.2) | 19735677.7 (18920371.7-20580350.4) | 24205.6 (23254.7-25194.4) | -1.01 (-1.05 to -0.97) |
| Tropical Latin America | 58726397 (55256854.9-62407938.3) | 37996.3 (35895.9-40380.3) | 51846393.7 (47957201.4-55694676.5) | 22995.9 (21309.4-24743.7) | -1.67 (-1.7 to -1.65) |
| Western Europe | 31325135 (29207067.5-33800038.5) | 8324 (7774.9-8963.7) | 27764098.9 (25925376.1-30162538.1) | 5987.3 (5563-6531.7) | -0.88 (-1 to -0.76) |
| Western Sub-Saharan Africa | 107840088.1 (105505627.8-110452942.6) | 52720.3 (51569.5-53927.2) | 198001823.7 (192301049.4-204421978.2) | 38270 (37335.9-39318.1) | -1.04 (-1.06 to -1.02) |
| Global | 1765110763.3 (1735880718.1-1794376239.5) | 32217.9 (31693.6-32740.9) | 1845246558.1 (1811946448-1882683058.9) | 23859 (23445.8-24320.8) | -0.98 (-0.99 to -0.96) |
| High SDI | 79096229.4 (75591321.4-82908356.3) | 9118.7 (8732.9-9522.9) | 74228336.8 (70673385-77972862.2) | 6458.8 (6173.1-6781.3) | -0.91 (-1.02 to -0.81) |
| High-middle SDI | 215302591.6 (208375347.5-221905661.4) | 20406.6 (19771.8-21028.3) | 156142863.9 (150977524.3-162351321.3) | 12006.9 (11625.1-12455.6) | -1.77 (-1.81 to -1.72) |
| Low SDI | 321458651.8 (316898242.4-325886013.5) | 62945.5 (62063-63775.3) | 496951265.7 (486858432.5-506781211.8) | 44208.9 (43375.1-45081.2) | -1.2 (-1.27 to -1.13) |
| Low-middle SDI | 612148578.4 (600041562-624013702.1) | 51757.3 (50783.7-52700.6) | 657750319.1 (644770853.9-671669215) | 34604.4 (33967.9-35312.8) | -1.31 (-1.32 to -1.29) |
| Middle SDI | 535577105.9 (522058155.5-549984340.9) | 30487.5 (29726.7-31238.9) | 458836863.7 (447289271.5-471001855.1) | 19136.4 (18700.2-19617.5) | -1.48 (-1.5 to -1.46) |
| **Incidence** | | | | | |
| Andean Latin America | 4324050.3 (3968542-4719109.4) | 10347 (9529.5-11252.2) | 3300571.9 (3023871.2-3603401.5) | 4975.4 (4562.5-5432.1) | -2.7 (-2.94 to -2.46) |
| Australasia | 189488.9 (160872.1-226058) | 915.8 (777-1096.2) | 301024.4 (265789.4-353778.4) | 845.6 (741.3-996.5) | 0.03 (-0.18 to 0.24) |
| Caribbean | 4260122.5 (4016037.4-4507336.9) | 11640 (10974.1-12278.4) | 3033764 (2804570.9-3295404.2) | 6591.5 (6080.1-7179.4) | -2 (-2.06 to -1.94) |
| Central Asia | 6498243.3 (6075992.5-6929209.7) | 8894.2 (8339.6-9462.2) | 4749728 (4423462.1-5095799.1) | 4927.2 (4597.2-5282) | -1.9 (-2.04 to -1.75) |
| Central Europe | 21044355.2 (20102527.3-22174740.9) | 17286.9 (16504.8-18203.5) | 8321411.5 (7862903.6-8811423.6) | 7479.7 (7086.4-7915.7) | -2.81 (-2.92 to -2.7) |
| Central Latin America | 23202043.9 (21606348-24999915.6) | 13013.4 (12134.3-13918) | 13940959.7 (12822617-15237963.6) | 5594.3 (5148-6102.8) | -2.55 (-2.64 to -2.47) |
| Central Sub-Saharan Africa | 22761326.8 (21351920.9-24189195.6) | 36820.9 (34736-38886.9) | 34376562.3 (31334296.6-37468949.3) | 23067 (21152.9-24805.3) | -1.4 (-1.83 to -0.97) |
| East Asia | 145728245 (128205577.1-164193030.7) | 11535.3 (10239.4-12956.9) | 47953796.3 (42789520.5-53591197.8) | 3450.6 (3075.5-3859.5) | -3.7 (-3.83 to -3.57) |
| Eastern Europe | 4057740.9 (3573016.7-4615030.3) | 1891.2 (1660.1-2160.5) | 2171094.5 (1837607.9-2532988.7) | 1146.3 (948-1355.5) | -1.25 (-1.42 to -1.09) |
| Eastern Sub-Saharan Africa | 96784365.2 (94113250.5-99206370.1) | 49502.7 (48371-50624.1) | 93965430.9 (90500597.2-97701700.4) | 21823.7 (21112.3-22493.6) | -2.81 (-3.01 to -2.62) |
| High-income Asia Pacific | 3694594.8 (3257538.6-4225775.6) | 2330.4 (2051.3-2650.7) | 2546369.7 (2219509-2972885.4) | 1384.1 (1214.6-1602.1) | -1.46 (-1.59 to -1.34) |
| High-income North America | 5378090.5 (4581285.3-6312367.9) | 1931.2 (1651-2254.5) | 5871693.1 (4891130.8-7010324.4) | 1442.2 (1218.3-1695.9) | -0.76 (-1.05 to -0.48) |
| North Africa and Middle East | 51639850.6 (49635137.6-53818814.5) | 14294.8 (13771.4-14832.4) | 34091907.2 (32339076.8-36111543.5) | 5480.2 (5202.9-5800.6) | -2.94 (-3.08 to -2.79) |
| Oceania | 1339378.5 (1252387.9-1429921) | 18443.4 (17325.9-19591.9) | 1586341.7 (1451597-1741349.1) | 10329 (9522.3-11202.9) | -1.52 (-1.67 to -1.38) |
| South Asia | 332392142.9 (306306678-356480484.5) | 28292.5 (26126.1-30300.7) | 166181992 (150406213.8-183692446.5) | 9172 (8326.9-10138.3) | -3.59 (-3.83 to -3.35) |
| Southeast Asia | 103870039.7 (97630098.2-110410060.1) | 19895.1 (18775.3-21118.1) | 38912936.3 (36375563.5-41989770) | 5892.6 (5501.5-6347.4) | -3.74 (-3.83 to -3.66) |
| Southern Latin America | 5534433.9 (4999688.9-6146388) | 11027.3 (9963.2-12266) | 4151693.4 (3685485.3-4574474) | 6323.9 (5561.4-6960.6) | -1.67 (-1.92 to -1.43) |
| Southern Sub-Saharan Africa | 9818704 (9107990.7-10632702.8) | 15651.8 (14539.8-16853.8) | 6196018.3 (5683778-6800899.8) | 7473 (6864.3-8169.9) | -2.2 (-2.29 to -2.11) |
| Tropical Latin America | 36479450.2 (33271237.6-40093411.1) | 23477.8 (21509.4-25637.8) | 22986967.8 (20379716.5-26123602.3) | 10148.8 (8997.3-11433.6) | -2.78 (-2.87 to -2.69) |
| Western Europe | 8775025.3 (8051813.6-9684894.3) | 2386.8 (2195.9-2632.5) | 8279769.8 (7236188.5-9518797.5) | 1681 (1481.1-1911.4) | -0.5 (-0.72 to -0.29) |
| Western Sub-Saharan Africa | 77110003.4 (74778091.8-79502456) | 36033.1 (34996-36992.6) | 83209354.4 (79910374.4-86670835.5) | 15398.4 (14847.8-15975.9) | -2.72 (-2.81 to -2.63) |
| Global | 964881695.8 (928560815.2-1001236464.5) | 17112.5 (16470.3-17731.4) | 586129387 (562138520.2-614585958.3) | 7725.1 (7404-8109) | -2.52 (-2.67 to -2.38) |
| High SDI | 24546126.1 (22463966-26835800.8) | 2909.5 (2668-3180.9) | 19575039.7 (17266354.9-22245938) | 1671.7 (1484.7-1885.6) | -1.42 (-1.59 to -1.26) |
| High-middle SDI | 87788129 (81809640.1-94442968.6) | 8339.4 (7781.3-8950.1) | 41476684.1 (38159757-45288134) | 3305.3 (3046.4-3589.9) | -2.87 (-2.96 to -2.78) |
| Low SDI | 230001057.1 (223728220.3-236755376.1) | 42587.6 (41451.3-43795.2) | 225651527.1 (218004943.9-233508667.9) | 19047.6 (18448.1-19697.1) | -2.67 (-2.89 to -2.45) |
| Low-middle SDI | 351995174.6 (334313603.3-368810119) | 27944.9 (26526.4-29160.8) | 181642679.4 (171302453.7-193219091.5) | 9389.3 (8870.9-9951.1) | -3.48 (-3.64 to -3.32) |
| Middle SDI | 269727153.5 (256230128.4-284449669.8) | 14567.3 (13862.8-15328.3) | 117311987 (110221589-125794282.8) | 5016.3 (4705.3-5381.2) | -3.28 (-3.38 to -3.18) |
| **Mortality** | | | | | |
| Andean Latin America | 6898.8 (5966.8-7965.7) | 21.8 (19.4-24.5) | 2984.7 (2470.5-3615.3) | 5.2 (4.3-6.3) | -4.95 (-5.19 to -4.7) |
| Australasia | 84.4 (76.5-90) | 0.4 (0.4-0.4) | 164.3 (136.7-182.1) | 0.3 (0.2-0.3) | -1.62 (-1.92 to -1.32) |
| Caribbean | 4001.3 (3354.3-4789.3) | 11.1 (9.5-13.1) | 1881.3 (1516.7-2389) | 4.1 (3.3-5.4) | -3.04 (-3.42 to -2.65) |
| Central Asia | 900.7 (824-987) | 1.2 (1.1-1.3) | 275 (239.2-317.6) | 0.3 (0.3-0.4) | -5.18 (-5.59 to -4.77) |
| Central Europe | 194.1 (180.5-209.5) | 0.2 (0.2-0.2) | 746.9 (679.2-809.8) | 0.4 (0.3-0.4) | 1.89 (1.32 to 2.47) |
| Central Latin America | 25568.1 (24495.3-26672.3) | 25.6 (24.5-26.4) | 11380.4 (10189.3-12676.5) | 4.9 (4.4-5.5) | -5.43 (-5.53 to -5.33) |
| Central Sub-Saharan Africa | 23987.7 (17721.3-34877) | 35.2 (28.2-47.5) | 9213.4 (6450.2-12630.4) | 10.1 (7.5-13.3) | -4.24 (-4.58 to -3.9) |
| East Asia | 38161.3 (33059.1-43797.9) | 5.4 (4.8-6.1) | 16451.9 (13634.6-19382.5) | 1.1 (0.9-1.3) | -8.07 (-10.13 to -5.97) |
| Eastern Europe | 1105.5 (1066.3-1142.7) | 0.5 (0.5-0.5) | 877.8 (816.7-938.2) | 0.3 (0.3-0.3) | -3.12 (-4.1 to -2.13) |
| Eastern Sub-Saharan Africa | 119297.9 (93821.6-156123.4) | 58.2 (48.8-70.6) | 42359.7 (33760.6-51309.2) | 14.1 (12.1-16.2) | -4.51 (-5.4 to -3.61) |
| High-income Asia Pacific | 1074.9 (982-1141.3) | 0.6 (0.6-0.7) | 2443.1 (2017.8-2703.2) | 0.4 (0.4-0.5) | -1.26 (-1.4 to -1.12) |
| High-income North America | 2369 (2080.7-2513.3) | 0.7 (0.6-0.7) | 13502 (11300.6-14710.9) | 1.8 (1.6-2) | 2.51 (1.64 to 3.38) |
| North Africa and Middle East | 14165.7 (11134.8-20219.2) | 4.1 (3.4-5.3) | 5275.4 (4462.1-6226.7) | 1.3 (1.1-1.5) | -3.87 (-4.04 to -3.7) |
| Oceania | 238 (190.1-296.9) | 7.6 (6.3-9.1) | 292.8 (227.1-378.4) | 4.4 (3.6-5.4) | -1.84 (-1.88 to -1.79) |
| South Asia | 214969.2 (174957.8-260196.5) | 19.5 (16-23.2) | 35228.8 (29438.3-41649.8) | 2.6 (2.2-3) | -6.23 (-6.4 to -6.06) |
| Southeast Asia | 41160.4 (34177.9-49603.6) | 15 (12.6-17) | 29017.8 (24951.7-32476.3) | 6 (5.1-6.6) | -2.8 (-2.94 to -2.66) |
| Southern Latin America | 1944 (1852.7-2038.7) | 4.3 (4.1-4.5) | 1490.7 (1314.9-1618.4) | 1.7 (1.5-1.8) | -2.96 (-3.59 to -2.33) |
| Southern Sub-Saharan Africa | 7449.1 (6268.4-8988.7) | 13 (11.3-15.1) | 5743.1 (4639.5-6998.9) | 8.3 (6.8-10) | -0.81 (-1.04 to -0.58) |
| Tropical Latin America | 12776.7 (11837.9-13783.2) | 11 (10.3-11.7) | 6224.4 (5452.6-6712.5) | 2.6 (2.3-2.8) | -4.7 (-4.99 to -4.41) |
| Western Europe | 3581.7 (3167.9-3860) | 0.7 (0.6-0.7) | 8620.1 (6953.6-9697.3) | 0.7 (0.5-0.7) | 0.01 (-0.17 to 0.19) |
| Western Sub-Saharan Africa | 50190.8 (37720.1-68989.6) | 19 (15.2-24.3) | 28100.6 (20057.5-36319.5) | 6.4 (5.1-7.8) | -3.42 (-3.54 to -3.3) |
| Global | 570119.2 (485777-693881.1) | 10.9 (9.4-13) | 222274.2 (199730.7-247630.3) | 3 (2.7-3.4) | -4.41 (-4.84 to -3.98) |
| High SDI | 7797.3 (7002.5-8275.2) | 0.8 (0.7-0.8) | 24537.4 (20286.8-26825.9) | 0.9 (0.8-1) | 0.21 (-0.28 to 0.71) |
| High-middle SDI | 17140 (15542.2-18831.6) | 2.1 (1.9-2.3) | 13681 (11882.5-15158.7) | 0.8 (0.7-0.9) | -3.26 (-3.58 to -2.95) |
| Low SDI | 207305.1 (161460.4-270528.3) | 35 (28.9-43.1) | 78100.7 (61627.9-95120) | 8.7 (7.4-10) | -4.36 (-4.98 to -3.73) |
| Low-middle SDI | 227993.8 (191097.6-275044.4) | 19.6 (16.6-23) | 52233.7 (46201.8-58901.9) | 3.7 (3.3-4.1) | -6.05 (-6.72 to -5.37) |
| Middle SDI | 109572 (101307.9-119935.4) | 9.9 (9.1-10.6) | 53543.1 (48303.7-57698.5) | 2.6 (2.3-2.8) | -4.26 (-4.32 to -4.19) |
| **DALYs** | | | | | |
| Andean Latin America | 625218.2 (518413-744687.2) | 1396.6 (1181.1-1646.6) | 237065.2 (182190.5-305227.5) | 376 (290.9-481.6) | -4.48 (-4.67 to -4.29) |
| Australasia | 14043.2 (9188.7-20922.7) | 69.5 (44.7-106.2) | 17763.4 (11730.1-28237.7) | 53.4 (34-87.6) | -0.85 (-0.97 to -0.73) |
| Caribbean | 515706.5 (422279.2-635094.1) | 1323.5 (1080.5-1634) | 325160.4 (239143.3-437026.4) | 746.9 (553.6-1001) | -1.73 (-1.96 to -1.49) |
| Central Asia | 637552.6 (453032.2-885816.1) | 845.2 (595.8-1176.9) | 571576.1 (390226.2-816401.3) | 585.1 (399.9-837.1) | -1.49 (-1.61 to -1.37) |
| Central Europe | 428680.2 (283570.3-623528) | 360.3 (237.1-521.6) | 224407.9 (155096.1-323236.7) | 200.8 (134.9-293.5) | -2.04 (-2.13 to -1.95) |
| Central Latin America | 1758748.5 (1587513-1987773.9) | 1037.9 (948.9-1154.8) | 712763.1 (576156.2-897813.4) | 303.3 (246.9-380) | -3.94 (-4.05 to -3.83) |
| Central Sub-Saharan Africa | 2536795.3 (1949159-3505987.1) | 3065.6 (2429.4-3991.7) | 1475531.8 (1077597.7-1999448.5) | 1004.3 (738.8-1354.8) | -3.76 (-4.05 to -3.47) |
| East Asia | 6372105.2 (5103033.9-8191738.2) | 570.8 (462.2-725) | 2398822.1 (1637228-3456189.8) | 160.8 (110.5-228.3) | -6.17 (-7.52 to -4.8) |
| Eastern Europe | 774336.8 (527315.1-1115780) | 353 (242.2-508.1) | 481403.9 (337108.3-685573.3) | 236.5 (163.1-337.2) | -1.63 (-1.82 to -1.44) |
| Eastern Sub-Saharan Africa | 11426982.7 (9185969.3-14528584.9) | 4027.1 (3326.1-5033.9) | 5735279 (4535286.6-7231086.8) | 1203.6 (961.9-1502.2) | -3.87 (-4.57 to -3.16) |
| High-income Asia Pacific | 146203.8 (94881.9-223583.3) | 86.2 (56-133.6) | 154630.3 (107984.4-219002.9) | 60.3 (40.1-91) | -1.03 (-1.16 to -0.89) |
| High-income North America | 223783.1 (146083.2-343976.6) | 74.5 (48.1-115.7) | 639568.5 (478816.5-841149.7) | 137 (99.2-185.5) | 2.2 (1.94 to 2.47) |
| North Africa and Middle East | 3383308.1 (2615337.2-4436261.8) | 844.9 (644.1-1118.3) | 2801435.3 (1987898.8-3873220.4) | 455.8 (325.8-629) | -2.04 (-2.09 to -1.99) |
| Oceania | 53516.2 (40031.9-71376.4) | 776.5 (583.5-1037.6) | 88158.3 (61800.5-132057.6) | 610.4 (433.7-897.4) | -0.54 (-0.62 to -0.46) |
| South Asia | 35096797.8 (28574752.4-44285658.3) | 2796.9 (2246.6-3574.3) | 20778260.7 (14607575.5-28656309.1) | 1187.9 (840.7-1627.6) | -2.69 (-2.72 to -2.67) |
| Southeast Asia | 5143487.5 (4134118.9-6512203) | 1105 (899.4-1380.2) | 3255846.7 (2427928.9-4327584.1) | 505.6 (384.3-664.8) | -2.49 (-2.58 to -2.4) |
| Southern Latin America | 158223 (131462.9-196834.4) | 320.9 (267.4-398.1) | 78615.1 (54851.1-115525.1) | 116.3 (79.4-172.1) | -3.2 (-3.4 to -3) |
| Southern Sub-Saharan Africa | 992199.7 (821072.4-1205771.4) | 1554.3 (1276.7-1910.1) | 866524.2 (684643.7-1089205.3) | 1089.9 (865.1-1364.7) | -0.64 (-0.83 to -0.46) |
| Tropical Latin America | 1760950 (1445446.4-2164069.3) | 1131.7 (940.4-1387.2) | 968432.9 (685625.1-1330149.8) | 446.6 (315.6-611.6) | -3.16 (-3.27 to -3.05) |
| Western Europe | 386945.9 (259996.8-577479.6) | 105.9 (69.9-158) | 487326.7 (328125.8-721784.7) | 94.6 (59.2-138.8) | -0.11 (-0.27 to 0.04) |
| Western Sub-Saharan Africa | 6238639.8 (4995123.8-8008122.3) | 2149.1 (1728-2711.3) | 6620689.5 (4927380.8-8770739.5) | 1109.8 (820.8-1479.6) | -2.15 (-2.23 to -2.07) |
| Global | 78674224 (64859006.9-97375904.4) | 1367.2 (1126.3-1708.5) | 48919261.1 (35986110.5-64985437) | 657.6 (489.9-869.6) | -2.52 (-2.71 to -2.32) |
| High SDI | 1109206.1 (782905.7-1605007.1) | 131.1 (92.5-189.7) | 1517919.3 (1120271.3-2055319.6) | 118.3 (83-165.1) | -0.07 (-0.17 to 0.04) |
| High-middle SDI | 4316931.7 (3243551.9-5836365.5) | 429.2 (326.5-575.1) | 2592001.3 (1799257.6-3685987.8) | 203.4 (140.8-289) | -2.64 (-2.73 to -2.54) |
| Low SDI | 23628234.3 (19283997-29778930.5) | 3334.2 (2727.8-4117.7) | 16170548.9 (12342103.5-20971971.2) | 1319.3 (1002.4-1722.9) | -2.97 (-3.31 to -2.62) |
| Low-middle SDI | 32747124.7 (26960541.9-40530875.8) | 2374.1 (1965.7-2980.9) | 18094875 (13077187.1-24551129.7) | 971 (706.7-1308.7) | -3.17 (-3.41 to -2.93) |
| Middle SDI | 16827000.8 (13719007.7-21383808.4) | 972.8 (797.3-1229.5) | 10513020.4 (7538644.2-14390371.4) | 459.5 (331.6-624.7) | -2.39 (-2.42 to -2.35) |
